# hMSC exosomes as a novel treatment for female sensitive skin: An *in vivo* study

**DOI:** 10.3389/fbioe.2022.1053679

**Published:** 2022-10-21

**Authors:** Congxiu Ye, Yunqing Zhang, Zhen Su, Shuxia Wu, Yuxia Li, Jinling Yi, Wei Lai, Jian Chen, Yue Zheng

**Affiliations:** ^1^ Department of Dermato-venereology, The Third Affiliated Hospital of Sun Yat-sen University, Guangzhou, Guangdong, China; ^2^ AIE Bioscience (Guangdong) Co., LTD., Torch Development Zone, Zhongshan, Guangdong, China

**Keywords:** hMSC exosomes, sensitive skin, regulating dermic immunity, suppressing neurovascular hyperreactivity, repairing skin barriers, biocompatibility, biodegradability

## Abstract

**Background:** Recent studies have reported that the incidence of sensitive skin is increasing. Skin sensitivity and skin barrier functions were related to many skin diseases including atopic dermatitis, psoriasis, rosacea, and so on. Mesenchymal stem cell (MSC)-derived exosomes (hMSC) might be considered as a new effective therapeutic scheme.

**Aims:** This study aims to investigate the safety and efficacy of hMSC exosomes as a novel topical treatment for sensitive skin.

**Patients/Methods:** Exosomes were extracted from primary hMSC *via* ultracentrifugation method. The morphology of hMSC exosomes was studied *via* transmission electron microscope. Expression of exosome specific surface marker was detected *via* Western blot. 22 subjects (female, aged 18–55) diagnosed with sensitive skin were enrolled. Follow-up was conducted before, 7-day, 14-day, and 28-day after hMSC exosomes use. Transepidermal water loss (TEWL), surface hydration, sebum secretion, and L*a*b* value were simultaneously tested at the same time point in an environment-controlled room.

**Results:** Under transmission electron microscopy, the extracted hMSC exosomes were circular or elliptical with intact membrane structure, and their diameters ranged mainly from 40 to 80 nm. Western blot showed that the expression of markers CD63, CD9, and Tsg101 was positive. Brownian motion based nanoparticle trajectory analysis (NTA) showed that the main peak of particle size distribution occurred around 96 nm, the average particle size was 122 nm, and the main peak accounted for 96.7%. All this conformed to the biological characteristics of exosomes standardized by the International Society for Extracellular Vesicles. In the clinical trial, scores of objective symptoms including roughness, scales, erythema, and subjective symptoms including tension, burning, or itching, were improved after 7-, 14-, and 28- day using hMSC-exosomes. TEWL, hydration, sebum, pH, and a* values were tended to return to the level of healthy skin.

**Conclusion:** The hMSC-exosomes, with the advantages of biocompatibility and biodegradability, could improve clinical symptoms and eruptions in sensitive skin patients, and might be as an MSC cell-free novel therapy in sensitive skin-related disease treatment.

## Introduction

Skin sensitivity was related to many skin diseases including atopic dermatitis, psoriasis, rosacea, and so on. Sensitive skin is a condition of subjective cutaneous hyper-reactivity to environmental factors such as cold, heat, and wind, and/or frequent or prolonged applications of some topical products, such as cosmetics (Berardesca, Farage, & Maibach, 2013). Recently, epidemiological studies found that sensitive skin was present in 56.8% of Koreans (Kim, Cheon, Misery, Taieb, & Lee, 2018). In China, the mean prevalence of sensitive skin was 13% (Yu et al., 2022). The condition was disturbing and detrimentally influenced the quality of life.

Extracellular vesicles (EVs), such as exosomes have been identified as mediators of an intercellular communication system. Exosomes contain a wide variety of proteins and nucleic acids that enable multifactorial signaling (Kalluri & LeBleu, 2020). Although MSCs have been used for a long time in tissue and organ repair (Akyurekli et al., 2015), EVs of mesenchymal stem/stromal cells (MSCs) have been found to promote comparable therapeutic activities as MSCs themselves. MSC-derived extracellular vesicles (EVs), which include exosomes and microvesicles (MV), are being replaced for MSC role in MSC cell-free therapies, owing to their biocompatibility, biodegradability, non-toxicity, and some specific therapeutic activities.

The therapeutic efficacy of MSC-EVs in various diseases, in diabetes mellitus, myocardial infarction, transplantation, cancer, macular degeneration, bone repairing, osteoarthritis, Alzheimer’s disease (AD), ischemic stroke, multiple sclerosis, as well as COVID-19 has been reported in clinical trials (Birtwistle, Chen, & Pollock, 2021). HMSC-exosomes (hMSC-exosomes) exhibit cardio and renal-protective activity, and are efficacious in animal models of myocardial infarction, stroke, brain injury (Ahn et al., 2021), and ischemia-reperfusion injury (Alzahrani, 2019). MSC-derived exosomes could be a novel therapeutic strategy for diabetic complications involving salivary glands. (AbuBakr, Haggag, Sabry, & Salem, 2020), ulcer foot (An, Chen, Tu, & Lin, 2021), Preclinical meta-analysis (Bailey et al., 2021), Kidney repair (Aghajani Nargesi, Lerman, & Eirin, 2017; Nargesi, Lerman, & Eirin, 2017), (Bai et al., 2018), acute renal injury (Birtwistle et al., 2021), also Inflammatory disease (Baharlooi, Azimi, Salehi, & Izad, 2020), such as Autoimmune Uveitis (Bai et al., 2017; Harrell et al., 2018; Shen et al., 2021) optic neuritis (Aneesh et al., 2021), and osteoarthritis (Bao & He, 2021). These suggested that hMSC-exosomes might be a compelling alternative to hMSCs in regenerative and aesthetic medicine, as they would avoid most of the problems associated with live MSC-based therapy (Golan et al., 2018; Pelizzo et al., 2018; Kalluri & LeBleu, 2020).

Zhang et al. (2015) found that wnt4 contained in umbilical cord mesenchymal stem cell-derived exosomes (HucMSC-Exo) may enhance the translocation and activity of *β*-catenin, thereby promoting the proliferation and migration of skin cells and angiogenesis. Fang et al. (2016) found that HucMSC-Exo could reduce scar formation and accumulation of myofibroblasts in a mouse model of skin defect. These functions are mainly dependent on the microRNAs contained in HucMSC-Exo. This revealed the potential effect of hMSC-exosomes in skin regional usage, but its safety and efficacy on sensitive skin has not been clarified until now.

In this study, we investigate the safety and efficacy of hMSC-exosomes as a novel topical treatment for sensitive skin, and preliminarily explore the possible biological mechanisms.

## Materials and methods

### Preparation of the formula

HFF-1 cells were purchased from Echo Biotech Co., Ltd., Beijing, China. CO_2_ incubator (Thermo Fisher, United States); refrigerated centrifuge (Sigma, United States); ultracentrifuge (Beckman, United States); transmission electron microscope (Hitachi, Japan); inverted phase contrast microscope (Olympus, Japan); flow cytometer (BD, United States); microplate reader (Molecular Devices, United States).

The umbilical cord was collected in a sterile bag and transported in a low-temperature transport box; Wharton’s jelly was separated from the umbilical cord within 48 h. The umbilical cord was placed in a 50 ml centrifuge tube, washed 3 times with gentamicin-containing PBS (final concentration of gentamicin: 25 ug/ml), and then washed 3–5 times with gentamicin-free PBS. About 0.5 cm was removed from each end of the umbilical cord and the blood was rinsed off. The umbilical cord was cut into 2–3 cm long sections that were then transferred to a 50 ml centrifuge tube. The umbilical cord pieces were cleaned and cut longitudinally along the intravenous cavity. The venous intima was peeled off and the two arteries were removed. The umbilical cord lining was fully removed and Wharton’s jelly was cut into small pieces about 3–5 mm long. They were then transferred into 150 mm dishes with each dish containing about 20 pieces and 10 ml of the medium. For storage, pieces can be placed in cryotubes and stored in a liquid nitrogen tank after programmed cooling. At 48 h, the dishes were supplemented with the medium (10 ml/dish). The medium was fully changed on the 7th and the 10th days. Mesenchymal stem cells crawled out from the 12th to the 14th days (or 15 to 18th days).

Third-generation hMSCs were tested using the human umbilical cord mesenchymal stem cell osteogenic differentiation medium kit according to the instructions for induction and differentiation. After 3 weeks of induction, the formation of mineralized nodules was examined under an optical microscope after Alizarin Red S staining. Third-generation hMSCs were tested with the human umbilical cord mesenchymal stem cell adipogenic differentiation medium kit according to the instructions to induce differentiation. When the lipid droplets became large enough and round, adipogenesis was examined under an optical microscope using Oil Red O staining. hMSCs were cultured for 48 h in serum-free mediums containing no exosomes. The cell supernatant was collected and centrifuged at 300 g for 10 min. The supernatant was then collected and centrifuged at 2000 g for 10 min. The supernatant was collected again, filtered with a 0.22 μm filter, transferred to an ultrafiltration centrifuge tube with 100 kDa MWCO, and centrifuged at 4,000 g for 30–50 min. The concentrated retentate was collected and centrifuged at 10,000 g for 30 min. The supernatant was transferred to an ultracentrifuge tube and centrifuged at 100,000 g for 70 min. The last step was repeated. All above centrifugation steps were conducted at 4°C. The obtained pellet consisted of exosomes, which was resuspended in PBS and stored at −80°C for later use. BCA kit was used to determine exosomal protein concentration.

The morphology of exosomes was studied using a transmission electron microscope. The sample-loading copper mesh was placed on filter paper, 20 μl of the exosomal extract was added dropwise, and the loaded mesh was let to stand at room temperature for 3 min 30 μl of 3% phosphotungstic acid solution was added for the negative staining that took 5 min at room temperature. Western blot was used to detect the expression of exosome specific surface markers CD63, CD81, and Tsg101. Exosomes were extracted and lysed in RIPA buffer and protein concentration was determined using the BCA method. SDS-PAGE electrophoresis was performed with 50 μg of sample per well and the proteins were transferred to a PVDF membrane by wet transfer method. Then the PVDF membrane was blocked with 5% skimmed milk powder for 1 h at room temperature, and anti-CD63, anti-CD9, anti-Tsg101, and anti-β-actin antibodies were added to the solution, which was incubated overnight at 4°C. HRP-labeled secondary antibody was added and the solution was incubated at room temperature for 30 min. ECL luminescent detection was conducted and recorded on a film. The cell culture medium (hMSC-Ed-CM) without exosomes was used as a control.

### Human fibroblasts scratch assay and cck-8

The scratch assay measures the migration ability of human fibroblasts. Straight lines were drawn on the back of a 6-well plate and human fibroblasts were inoculated. The appropriate density was for the cells to grow to full confluency overnight. After the cells reached full confluency, the cell monolayer was scratched with a 200 μl sterile pipette tip in lines perpendicular to the drawn lines. The plate was washed with PBS and replenished with the desired cell culture medium: containing 1% FBS for the CM group (cell culture control group) and final concentrations of 5, 10, 15, and 20 μg of exosome/ml for the Exo group to simulate the pathological environment of OA. Control groups were set up at the same time. Micrographs were taken at 0 and 12 h for each group. ImageJ software was used to analyze the scratch healing rates. CCK-8 kit detects the proliferation of human fibroblasts. Human fibroblasts were seeded into 96-well plates at a density of 2 × 10^3^ cells/well. The next day, each group was added with the desired complete medium (the experiment grouping was the same as before), and the culture was continued for 1, 3, 5, and 7 days. After that, 10 μl of CCK-8 solution was added to each well, incubation continued for 2 h, and the absorbance at 450 nm was measured with a microplate reader.

### Lactic acid

Lactic acid (Sigma Chemicals Co.) was prepared at a 5% concentration (w/v) in distilled water (DW).

### Subjects

22 healthy volunteers aged from 18 to 55, whose 5% lactic acid stinging test scores were ≥3 and had repeatedly dry, tingling, burning, itching, or other discomfort symptoms participated in this study after informed consent. Subjects who were pregnant, lactating, or planning to pregnant during the trial; who had pre-existing skin disease (e.g., acne, eczema, psoriasis) or were taking the anti-inflammatory drugs; who had undergone facial cosmetic surgery, facial lifting, wrinkle removal, or scar smoothing, or planned to reshape their face within 6 months before or during the trial were excluded from the study. The study was conducted in accordance with the tenets of the Declaration of Helsinki. The study protocol was approved by the Ethics Committee Board of the hospital (2020-008-01).

### Clinic study schedule

Before participation, volunteers received vital signs detection. Each volunteer visited the research center four times in total: prior to intake at baseline (0 D), 7, 14, and 28 days after the use of the product. Volunteers were given a two-week washout period prior to the formal trial, during which they stopped using their own facial moisturizers to exclude the effects of products used prior to participation in the trial. During the study, all volunteers applied 1 ml to the face twice a day (morning and evening), after thawing the product into a liquid state and stopped using their own facial moisturizers. After thawing or unsealing, the product should be stored at 2–8°C and used up within 24 h. Adverse events caused by the formula were monitored throughout the study *via* regular consulting and questionnaires.

Subjective assessment was conducted by the same dermatologist before, 7, 14, and 28 days after the use of the product, including the objective and subjective symptoms and the improvement index. The scoring criteria were as follows. Calculation of improvement index: improvement index = (pre-treatment score—post-treatment score)/pre-treatment score × 100%. According to the improvement index, it was divided into complete improvement (improvement index >90%), significant improvement (improvement index 60%–89%), improvement (improvement index 20%–59%) and ineffective (improvement index <20%).

0 points: not dry, scales, erythema, papules, and no subjective symptoms;
1 points: slightly dry and rough, slight scales and erythema, slight tension, burning, or pruritus;
2 points: moderately dry and rough, moderate scales and erythema with a small amount of papules, moderate tension, burning, or pruritus;
3 points: severely dry and rough, severe scales and erythema with obvious papules or pustules, severe tension, burning, or itching.

The lactic acid stinging tests were conducted before, 14, and 28 days after the use of the product according to the method described previously (Misery, 2017; Ye et al., 2020).

All subjects and environments were prepared according to the method described previously (Misery, 2017; Ye et al., 2020). The cheek, the main paroxysm site of sensitive skin, was selected as the instrumental test region. The TEWL, the skin surface hydration, the skin sebum, the skin surface pH value, and the skin a* value which indicates the color direction, and the increase of a* value indicates red, a decrease of a* value indicates the color direction toward green, were measured with the Tewameter^®^ TM 300, Corneometer®Derma, Sebumeter^®^ Derma, Skin-pH-meter^®^ Derma of Unit SSC3, and colorimetry CM-700d (KONICA MINOLTA), described with the L*a*b* system (Chardon, Cretois, & Hourseau, 1991)respectively, according to manufacturer’s instructions. The clinical photographs taken with a VISIA™ system (Canfield Inc.), were conducted before, 7, 14, and 28 days after the use of the product.

Volunteers filled out questionnaires at 7, 14, and 28 days after the use of the product to provide their subjective satisfaction with the product. The evaluation standard was divided into A-E levels: level A, dissatisfied; level B, somewhat satisfactory; level C, satisfactory; level D, very satisfactory; level E, extremely satisfactory.

### Statistical methods

Statistical analysis was conducted using SPSS^®^ 20.0 software (IBM International Business Machines Corporation). The data were compared using the paired comparison Student’s t-test. *p* < 0.05 was regarded as statistically significant.

## Results

### Cell isolation and characterization

**FIGURE 1:**
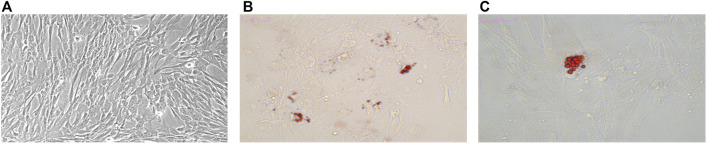
Characterization of hMSCs. Osteogenesis and adipogenesis induced differentiation of hMSCs. **(A)** Morphology of hMSCs. **(B)** Adipogenic differentiation observed with Oil Red 0 staining (× 200). **(C)** Adipogenic differentiation observed with Oil Red 0 staining (× 400).

The isolated primary human fibroblasts were stellate or short spindle-shaped (Figure 1A), had a high refractive index and looked like “pavement stones” when the density was high. They could be stained with toluidine blue. After adherence, hMSCs took a fusiform shape and grew in a whirlpool pattern.

### Characterization of exosomes

**FIGURE 2:**
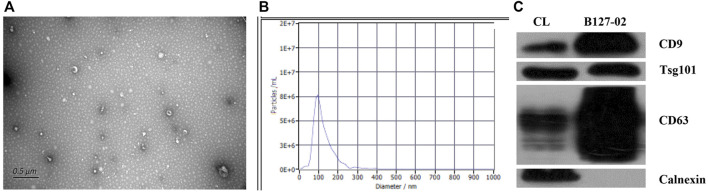
Characterization of hMSCs-exosomes. **(A)** Exosomes under transmission electron microscope **(B)** Brownian motion based nanoparticle trajectory analysis (NTA). **(C)** Western blot detection of the expression of exosomal surface marker proteins.

The isolated hMSCs were characterized by stem cell multi-differentiation experiments, and the results are shown in Figure 1. After osteogenic induction, calcium deposits gradually appeared in hMSCs, After adipogenic induction, hMSCs gradually changed from a spindle shape to a round shape, small lipid droplets appeared in the cells, and the lipid droplets gradually merged into larger ones. On the 14th day, the Oil Red O stained lipid droplets appeared red (Figures 1B,C). Under transmission electron microscopy, the extracted hMSC exosomes were circular or elliptical with intact membrane structure, and their diameters ranged mainly from 40 to 80 nm (Figure 2A). Western blot showed that the expression of markers CD63, CD9, and Tsg101 was positive (Figure 2C).

### Brownian motion based nanoparticle trajectory analysis

The main peak of particle size distribution occurred around 96 nm, the average particle size was 122 nm, and the main peak accounted for 96.7% (Figure 2B). All this conformed to the biological characteristics of exosomes standardized by the International Society for Extracellular Vesicles.

### Human fibroblasts migration and proliferation

**FIGURE 3:**
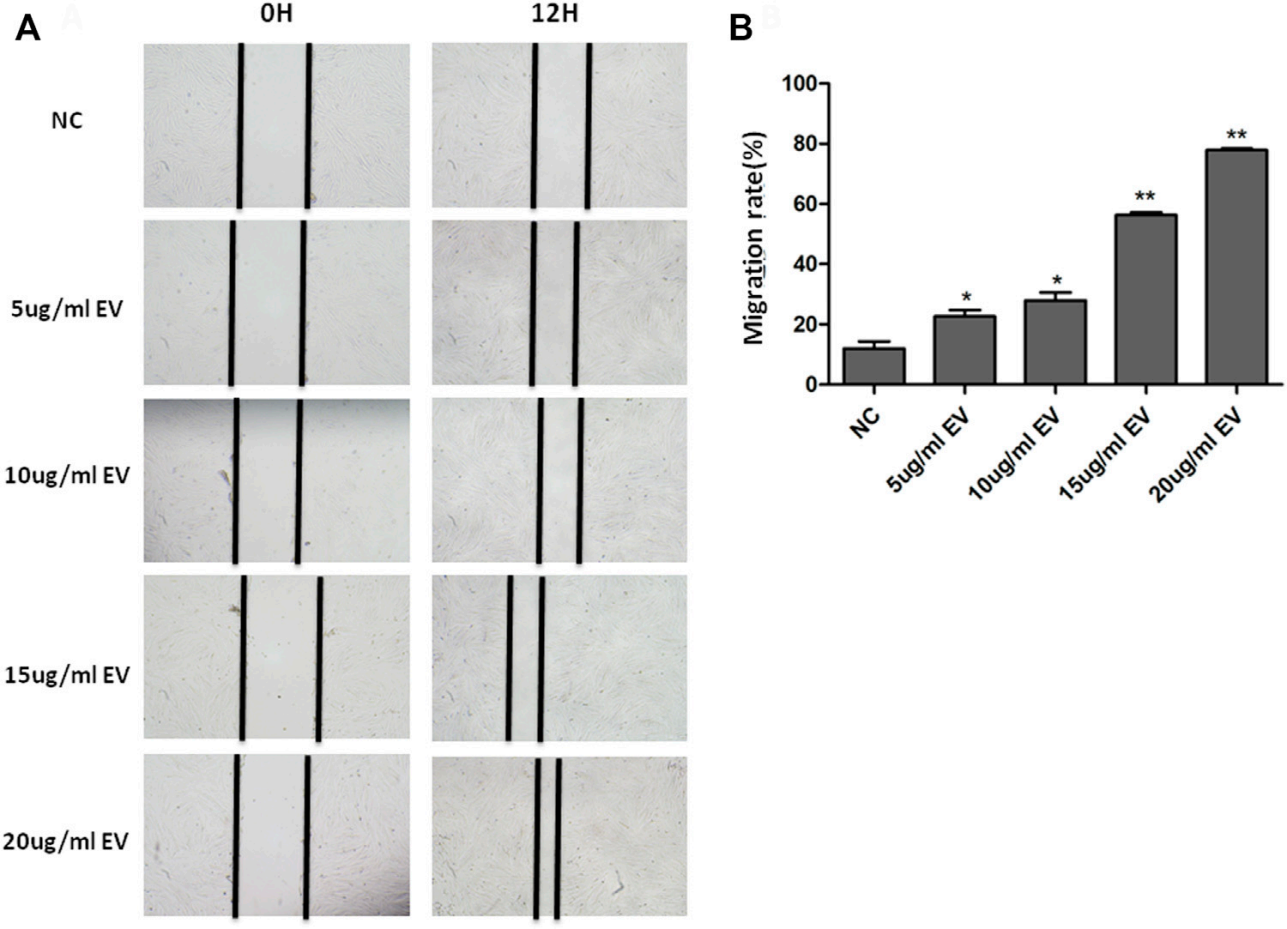
The effect of hMSC-Exo to human fibroblasts migration. **(A)** The microscopic features of human fibroblast with different concentrations of hMSC-Exo (5, 10, 15, and 20 μg/ml). **(B)** The migration rate of human fibroblast scratch assay. *p < 0.05, * *p < 0.01, compared to NC group.

The scratch assay showed that hMSC−Exo significantly promoted the healing of human fibroblast scratches, and the healing speed was significantly accelerated with the increase of hMSC−Exo concentration and prolonged action time (Figure 3A). It had almost completely healed when treated with hMSC−Exo (20 μg/ml) for 12 h. The scratch test showed that the proliferative ability of cells of exosome-treated group was enhanced compared with the NC group. The effect was dose-dependent (Figure 3B).

**FIGURE 4:**
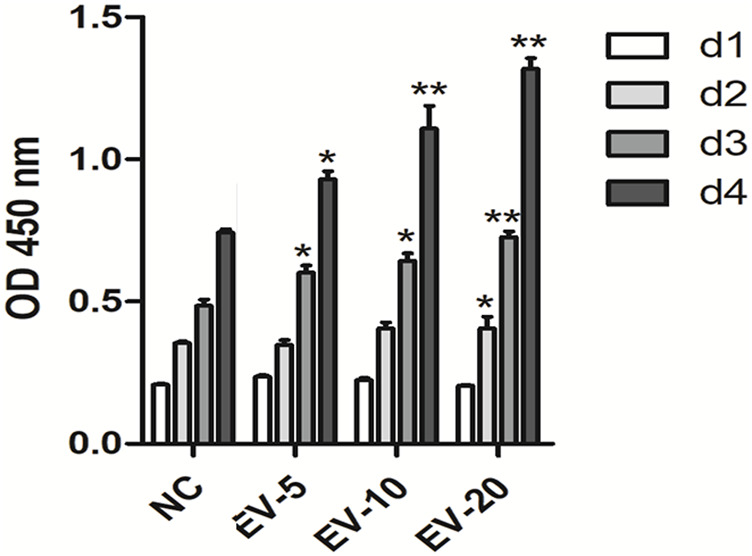
The effect of hMSC-Exo to human fibroblasts proliferation. *p < 0.05, **p < 0.01, compared to NC group.

The CCK-8 results showed that hMSC-Exo significantly promoted the proliferation of human fibroblasts in a time- and dose-dependent manner (*p* < 0.01) (Figure 4).

### Clinical trial result

A total of 22 healthy female subjects were included in the trial, ranging from 24 to 55, and the average age was 40 ± 8.07. Two of the subjects were lost to follow-up due to the epidemic situation. The other 20 subjects completed the clinical trial as required. At the end of the clinical trial, no adverse event was found in the volunteers.

**FIGURE 5:**
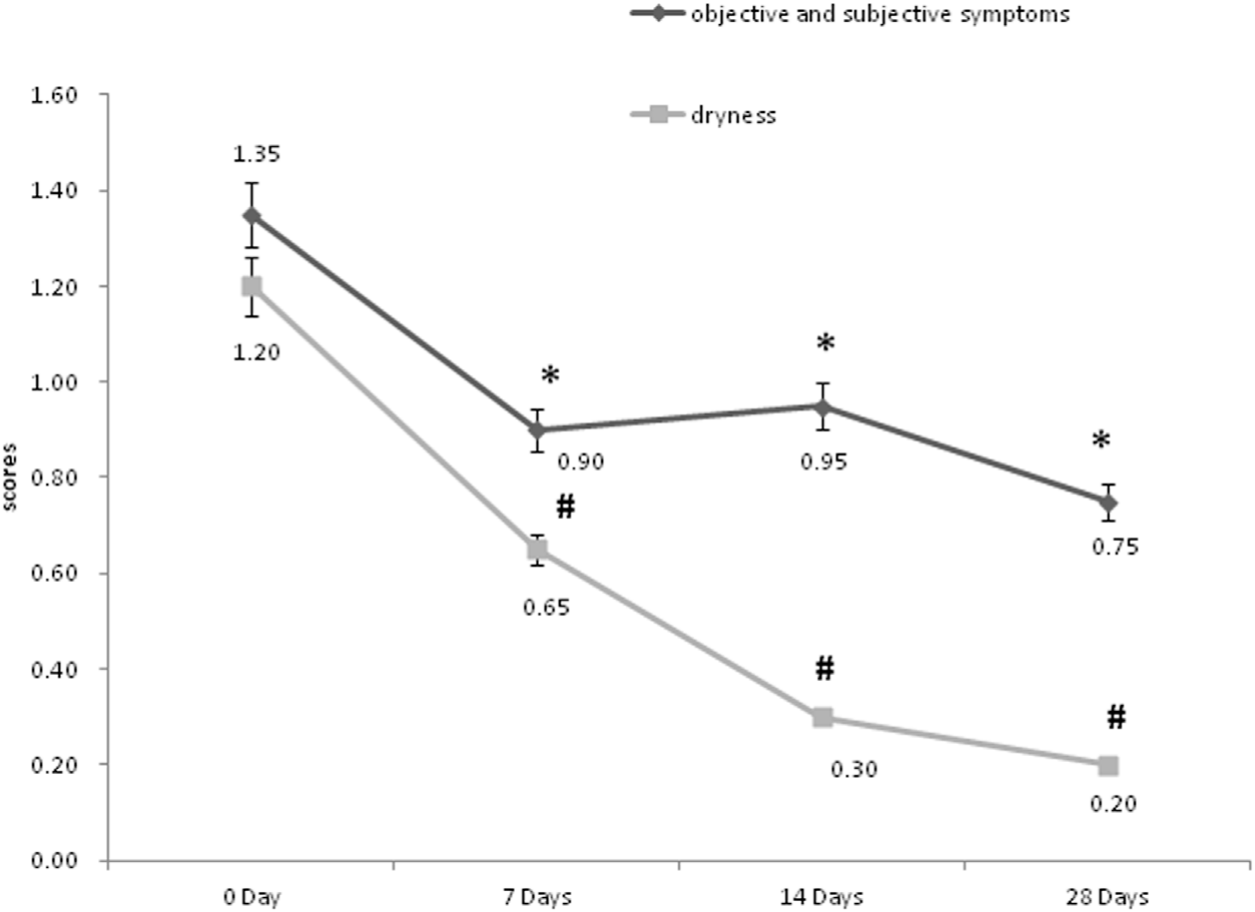
The objective, subjective symptoms and dryness assessment of the dermatologist. *p < 0.05, scores of objective and subjective symptoms, compared to the baseline (0 Day). #*p* < 0.05, scores of dryness, compared to the baseline (0 Day).

In order to assess the efficacy of the product for sensitive skin, the objective and subjective symptoms assessment was conducted by the same dermatologist firstly. As shown in Figure 5, scores of objective symptoms including roughness, scales, erythema, and subjective symptoms including tension, burning, or itching, both decreased significantly on 7, 14, and 28 days after the use of the product compared to the baseline (*p* < 0.05); scores of dryness decreased significantly on 7, 14 and 28 days after the use of the product compared to the baseline (*p* < 0.05). Then the improvement index was calculated. On 7, 14, and 28 days after the use of the product, the objective and subjective symptoms were all improved (33.3%, 29.6%, and 44.4% separately). The dryness symptoms showed improvement on day 7 (45.8%), and significant improvement both on day 14 and day 28 (75.0%, 83.3% separately).

**FIGURE 6:**
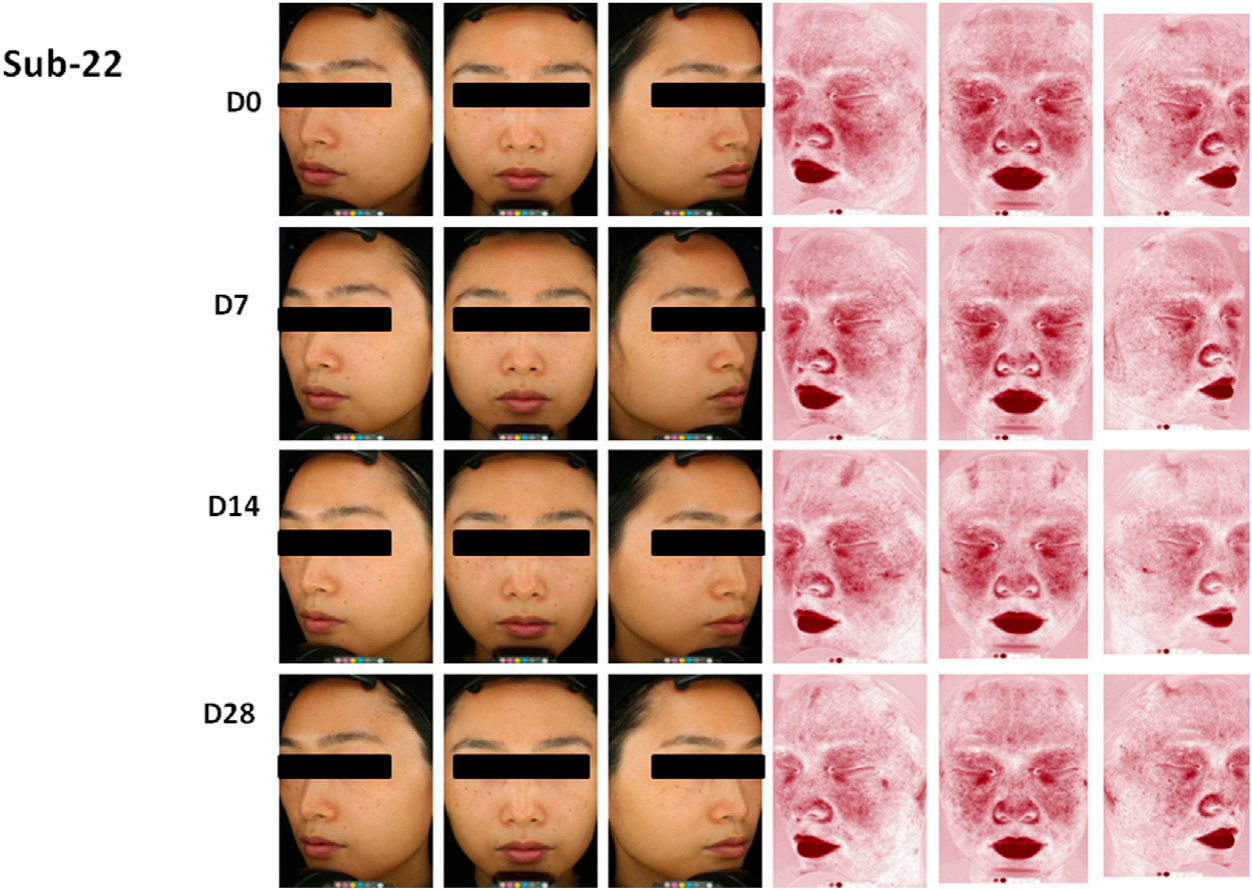
Photographs of volunteer #22 with VISTA” system at 0 day (DO), 7 days (D7), 14 days (D14) and 28 days (D28), Improvement of red areas could be seen at 28 days.

Secondly, the skin indexes of TEWL, hydration, sebum, pH value, and the skin a* value were measured, and data were shown in Table 1. The skin a* value, which is an important index of the efficacy of the product for sensitive skin, decreased significantly on 14 and 28 days after the use of the product compared to that at the baseline (*p* < 0.05). The lactic acid stinging test scores decreased significantly on 7, 14, and 28 days after the use of the product compared to the baseline (*p* < 0.05). The skin sebum decreased significantly on 14 days after the use of the product compared to that at the baseline (*p* = 0.026). The skin surface pH value increased significantly on 28 days after the use of the product compared to that at the baseline (*p* = 0.026). Although there was no significant difference between the skin TEWL and hydration at each time point after the use of the product compared to the baseline (*p* > 0.05), the skin TEWL and hydration decreased or increased gradually with the extension of the use time of the product. Figure 6 showed the clinical photographs of one volunteer (sub-22) using the VISIA™ system. At 28 days, when compared to baseline (0 days), the apparent alleviation of red areas was observed in the photographs.

**TABLE 1:** TEWL, hydration, sebum, pH, a* value, and lactic acid stinging test scores on 0,7, 14, and 28 days after the use of the product (mean ± SD).

Parameter	0 Day	7 Days	14 Days	28 Days
Hydration (AU)	76.34 ± 8.24	74.58 ± 9.13	73.11 ± 11.43	77.29 ± 9.56
TEWL (g/m2h)	12.62 ± 4.60	12.57 ± 4.87	12.26 ± 3.19	11.96 ± 3.17
Sebum (AU)	51.00 ± 29.68	46.35 ± 29.54	43.20 ± 30.72*	50.35 ± 32.41
pH value (AU)	6.05 ± 0.55	6.29 ± 0.51	6.06 ± 0.67	6.28 ± 0.53*
a* value (AU)	12.17 ± 2.23	9.94 ± 3.73*	9.15 ± 3.50*	11.56 ± 1.96*
lactic acid stinging test scores	8.83 ± 6.10	—	4.70 ± 4.17*	4.85 ± 4.75*

* *p* < 0.05, compared to the baseline (0 Day).

Thirdly, the proportions of subjects who are very satisfied with the product use experience *via* questionnaires were 75% on 7 days, 75% on 14 days, and 80% on 28 days.

## Discussion

Although the pathogenesis of sensitive skin is not fully understood, skin barrier damage, inflammation, blood vessels, nerve hyperresponsiveness, and vascular proliferation/dilation were identified as key mechanisms of sensitive skin by many studies. Soluble components of MSC exosomes were capable to promote tissue regeneration, and suppress detrimental immune responses and neuron regeneration in ischemic tissues. MSC-derived secretomes showed immunoregulatory, neuron-protection, and anti-apoptotic effects that resulted in enhanced tissue repair and regeneration (Ha et al., 2020). In previous experimental and preclinical settings, exosome-based therapies are widely investigated in various disease models. In this study, we found that the hMSC exosomes could improve clinical symptoms, skin eruptions, and skin indexes including TEWL, hydration, sebum, pH, and the L* a* value of sensitive skin patients. Therefore, we suggested that hMSC-exosomes could improve skin sensitive conditions by repairing skin barriers, regulating dermic immunity, and suppressing neurovascular hyperreactivity.

Sensitive skin is related to skin barrier dysfunction, which can lead to discomfort in various skin diseases (Roussaki-Schulze et al., 2005; Saint-Martory et al., 2008). Pinto et al. (2011) found that the TEWL (transepidermal water loss) of sensitive skin patients was significantly different from the normal control group by using the curve mathematical model analysis (Pinto, Rosado, Parreirão, & Rodrigues, 2011). Decreased neutral lipid levels and sheath phosphorus and the increase of lipid level in the skin of patients with sensitive skin reduces the stability of skin barrier function (Cua, Wilhelm, & Maibach, 1990; Pinto et al., 2011). The decline of barrier function is not only conducive to the entry of external irritants into the skin surface, make nerve endings more vulnerable to stimulation, and increase TEWL value (Saint-Martory et al., 2008) (Warren et al., 2005). In this study, we found that the skin index as TEWL and skin hydration which reflected the skin barrier function were improved after using hMSC-exosomes. For Hu et al. (2016) found that adipose-derived mesenchymal stem cell-derived exosomes (ASCs-Exo) could stimulate the proliferation, migration, and collagen synthesis of fibroblasts in a dose-dependent manner, and promote cyclin 1, N-calcium. The expression of mucin, collagen type I, collagen type III and proliferating cell nuclear antigen, thereby promoting the healing of skin wounds. This may partly explain the mechanisms of dermal and epidermal repairing effects of hMSC-exosomes on sensitive skin. We suggested that the regulatory roles of hMSC exosomes in recovering skin barrier functions might be by stimulating the skin cell activity and proliferation, and promoting the secretion of sebum which is one of the important components of normal skin barrier.

Our dates showed that the skin a* value was decreased after 7-day of treatment and the skin inflammatory red area was improved under VISIA which revealed that hMSC-exosomes had anti-inflammatory and therapeutic effects. The immune pathogenesis of sensitive skin is quite complicated, while the immune regulation functions of exosomes derived from different cell sources has been reported. Li et al. (2016) studied the effect of human umbilical cord mesenchymal stem cells (hUMSCs) on the inflammatory response in a diabetic rat burn model, and found that the endogenous miR-181c of hUMSCs exosomes can pass Inhibit Toll-like receptor 4 (TLR4) signaling pathway, attenuate lipopolysaccharide-mediated inflammatory response, reduce the number of inflammatory cells such as neutrophils and macrophages, and the expression of inflammatory factors such as TNF-α and IL-1β, and promote anti-inflammatory factors IL-10 expression, thereby inhibiting the inflammatory response. In addition, miR-155 encapsulated in exosomes of bone marrow MSCs can promote endotoxin-induced inflammatory response, while miR-146a inhibits inflammatory response, and the two synergistically regulate the expression of inflammatory genes (Alexander et al., 2015). Cha et al. showed tonsil-derived mesenchymal stem cells (T-MSCs) were able to effectively attenuate TLR7-mediated skin inflammation in mice, which was accompanied by an increase in mast cell number. The present study investigated whether T-MSC extracellular vesicles, such as exosomes, are able to regulate mast cell activation in response to TLR7 stimulation. (Cho et al., 2021). We suggested that hMSC might take part in adjusting multiple inflammatory immune factors, signaling pathways and non-coding RNAs in sensitive skin. And the characteristic immune mechanisms still need further investigation.

Cutaneous vascular hyperreactivity and neurosensory dysfunction can be present in sensitive skin patients both with or without erythema. Sustained chronic inflammation characterized by a mainly Th1 macrophage and mast cell-driven infiltrate further leads to the release of incompletely understood mediators involved in vasoregulation, immunity, and fibrosis (Mascarenhas, Wang, Chang, & Di Nardo, 2017). TRP channels hyper-activation, such as TRPA1, TRPV1, TRPV3, and PAR2, can crosstalk with neuropeptide receptors or at least trigger neuropeptide release (Aubdool & Brain, 2011; Steinhoff, Schauber, & Leyden, 2013; Mascarenhas et al., 2017). TRPV3 mediates reactions to warm temperatures and camphor. TRPV4 can be activated by heat, mechanical and hypo-osmotic stress as well as UV. This hyper-reactivity can be modulated by multiple environmental factors. The link between the therapeutic mechanism of exosome and TRPV inhibition has not been fully established. However, Li et al. (2020), showed that BMSCs-exosomes could relieve chronic pain *via* inhibition of the CGRP-positive nerve, a key effector of TRPV activation (Park et al., 2018). A study by Yu et al. (2020), found adipose-derived exosomes play a protective role in lung injury by alleviating the pulmonary endothelial barrier injury and inflammatory response, through inhibiting the TRPV4/Ca2+ pathway. Further *in vivo* data verified that TRPV4 and ROCK1 played a centric role in the neuropathic process (Yu et al., 2022). These suggested that hMSC-exosomes might improve clinical symptoms and eruptions by regulating dermic immunity and suppressing neurovascular hyperreactivity in sensitive skin patients. But its mechanisms still need further investigation.

## Conclusion

The hMSC-exosomes, with the advantages of biocompatibility and biodegradability, could improve clinical symptoms and eruptions in sensitive skin patients, and might be as an MSC cell-free novel therapy in sensitive skin-related diseases treatment.

## Data Availability

The raw data supporting the conclusion of this article will be made available by the authors, without undue reservation.
